# Have FDI quantity and quality promoted the low-carbon development of science and technology parks (STPs)? The threshold effect of knowledge accumulation

**DOI:** 10.1371/journal.pone.0245891

**Published:** 2021-01-25

**Authors:** Shujing Zhang, Beibei Hu, Xiufeng Zhang

**Affiliations:** 1 School of Management and Economics, North China University of Water Resources and Electric Power, Zhengzhou, China; 2 Institutes of Science and Development, Chinese Academy of Sciences, Beijing, China; 3 Business School, Henan Normal University, Xinxiang, China; China University of Mining and Technology, CHINA

## Abstract

In recent times, China has emphasized five major development concepts to promote high-quality development: coordination, green, innovation, openness, and sharing. As a metamorphosis of these ideas, Chinese science and technology parks (STPs) are gathering areas of high-tech industries and represent advanced productive forces. Their greenness, openness, and innovative developments herald the future development trends of China. Based on the data of 52 STPs in China from 2011 to 2018, this study analyzes the impact of foreign direct investment (FDI) quantity and quality on the low-carbon development of the STPs. We use Hansen’s nonlinear panel threshold regression model with knowledge accumulation as the threshold variable. The results show the following: First, there are complex nonlinear relationships between FDI quantity, FDI quality, and the low-carbon development of the STPs. Second, FDI quantity has a significant positive impact on the low-carbon development of the STPs only when the level of knowledge accumulation is below a certain threshold. Beyond this threshold the effect is no longer significant. Third, FDI quality has a significant positive impact on the low-carbon development of STPs only when the level of knowledge accumulation is lower than a certain threshold; beyond which, the impact is no longer significant. These results can serve as a reference for China to effectively promote economic low-carbon growth of STPs and achieve green, open, and innovative development.

## 1. Introduction

As the largest developing country in the world, China has made tremendous progress with 30 years of rapid economic growth: from the reformation and opening up in 1978 to the outbreak of the global financial crisis in 2008. In this process, the Chinese government promoted rapid economic growth by accelerating the development of high-tech industries and science and technology parks (STPs). These STPs have played a leading role in and greatly promoted China’s development [1, 2]. Meanwhile, the Chinese government has adhered to opening up to the outside world and bringing in foreign direct investments (FDI). FDI has brought a great amount of capital, technology, and experience, thereby greatly accelerating China’s economic and social development. This has profoundly promoted the knowledge and technological progress of high-tech industries and STPs. However, they have also resulted in industrial problems such as high pollution and high energy consumption [3–6]. The reasonable introduction and utilization of FDI to promote economic high-quality development has become an important issue in China in the new era. In view of these issues, China has intensified efforts to seek high-level opening up accompanied by high-quality development in the new stage of development. Toward the end of October 2015, the proposal involving green development in the “Thirteenth Five-year Plan” (2016–2020) was adopted at the Fifth Session of the 18th National Congress of the Communist Party of China. In this proposal, the five development concepts, “innovation, coordination, green, openness, and sharing” were first proposed. To show its concrete intentions, at the 21^st^ United Nations Climate Change Conference in 2015, China pledged to reduce its carbon intensity by 60%–65% (carbon dioxide emissions per unit of gross domestic product (GDP)) by 2030 compared to 2005. In other words, low-carbon and high-quality opening up have become important development goals for China in the current era.

**Fig 1:**
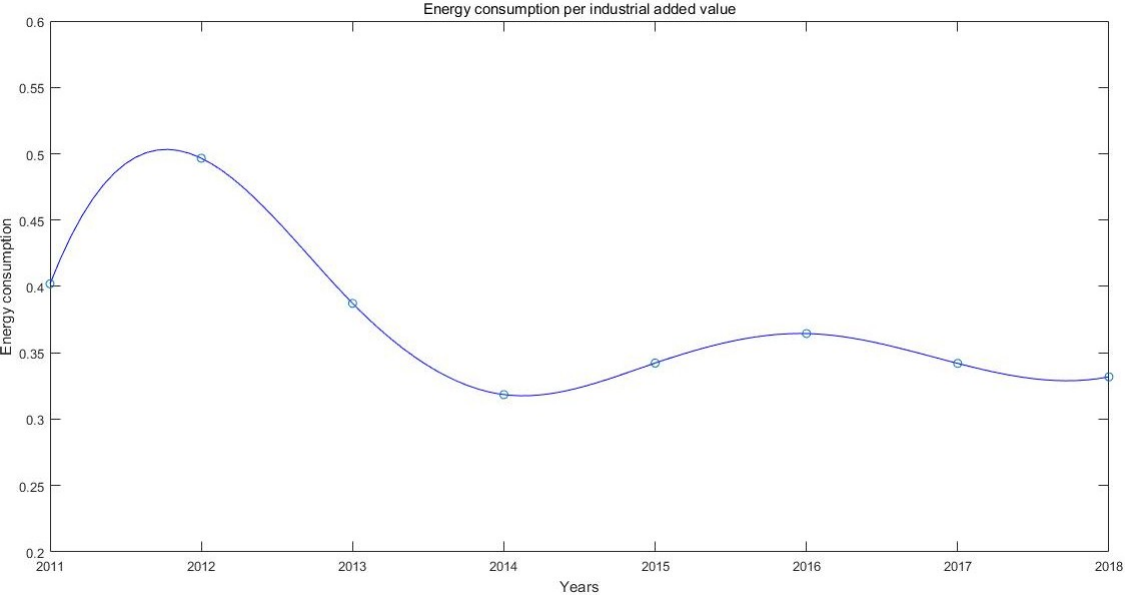
Change of energy consumption per unit industrial added value in the STPs.

To achieve high-quality, low-input, and low-energy development, the Chinese government has adopted a point-to-face approach to promote economic low-carbon growth, with national STPs being one of its core points. The construction of STPs is an important step for China to implement high-tech industrialization development. National STPs are the gathering areas of high-tech industries, representing China’s advanced technology and productivity. They serve as important platforms to promote technological innovation and transformation of scientific and technological achievements through independent innovation and the introduction of FDI. The future development goal of national STPs is to improve the level of technological innovation, transformation rate of achievement, and sustainable development. Therefore, reducing unit energy consumption and improving energy utilization are also requirements for the national STPs. According to the statistical data from 2011 to 2018, the energy consumption per unit of industrial added value of national STPs has shown a significant downward trend as a whole. This indicates that STPs have significantly improved energy utilization efficiency; that is, they are on track for achieving low-carbon development (Fig 1).

To promote and implement low-carbon development, STPs insist on opening up to the outside world, focusing on the introduction of FDI to improve the innovation capabilities and technological level of the enterprises in the park.

In 2018, the amount of FDI absorbed by enterprises in the STPs reached 3362.6 billion-yuan, accounting for 38.0% of the total FDI in the country. This shows the leadership of China’s STPs in fully utilizing FDI and promoting sustainable development. However, the impact of FDI on the low-carbon growth of STPs is more complex and is subject to several factors, such as their own knowledge accumulation and technological level. Studies show that the knowledge spillover due to FDI shows a non-linear effect because of the differences in the technological level or knowledge absorption capacity of the host country. A higher technological level or knowledge absorption capacity is conducive to improving the host country’s absorption of FDI knowledge spillovers [7, 8]. As the gathering areas of high-tech industries with characteristics of more research and development (R&D) investment and knowledge accumulation, national STPs’ level of knowledge accumulation often reflects their technological level and knowledge absorption capacity. Therefore, based on previous theoretical and empirical studies, one may argue that the impact of FDI on the low-carbon growth of STPs may also be subject to China’s technological level or knowledge accumulation and exhibit nonlinear characteristics. However, there are few studies on this topic.

This paper asks the following questions to address these aspects. As an important symbol of open development and an important external factor, has FDI actively and effectively promoted efficient energy utilization and low-carbon development of the STPs? If FDI plays an important role in low-carbon development, will it be affected by the level of knowledge accumulation of the STPs? That is, does the impact of FDI on the STPs’ energy utilization rate exhibit nonlinear characteristics due to their own knowledge accumulation? Are there differences between the impacts of FDI quantity and quality on the low-carbon development of the STPs? How can the government more effectively introduce FDI to promote low-carbon economic growth?

This study incorporates FDI, knowledge accumulation, and low-carbon development of STPs into the same research framework. Then, it explores the nonlinear relationship between FDI and low-carbon development under different levels of knowledge accumulation in STPs. The two main contributions of this study are as follows. First, this study contributes theoretically by providing evidence on the non-linear relationship between FDI and the low-carbon development of STPs. Second, this study can practically help China understand the specific mechanisms of FDI, knowledge accumulation, and low-carbon development. This can provide a basis for the country to reasonably and effectively formulate FDI policies and measures for the development of STPs.

The content of this paper is divided into the following parts. Section 2 presents the literature review on FDI and the development and performance of STPs. Section 3 constructs the threshold regression model, explains the data source, and variable selection. Section 4 uses the threshold regression model to analyze the impact of FDI quantity and quality on low-carbon development in STPs at different levels of knowledge accumulation. Section 5 presents the conclusions and provides policy recommendations.

## 2. Literature review and theoretical analysis

Current studies have extensively researched FDI, mainly focusing on the impact of FDI on the host country’s environmental pollution, and low-carbon development, let alone the impact of FDI on the STPsand the nonlinear relationship between the two. In the context of this paper, the literature review focuses on the following content: the impact of FDI on the host country’s economy, technology, energy consumption, and environmental pollution; the relationship between FDI and human resource accumulation and knowledge accumulation of the host country; and the impact of FDI on high-tech industries or high-tech enterprises.

### 2.1 FDI and host country’s environmental pollution and low-carbon development

There are two classic hypotheses regarding the relationship between FDI and the host country’s environmental pollution and low-carbon development: “pollution paradise hypothesis” and “pollution halo hypothesis.” The pollution paradise hypothesis posits that developed countries tend to keep high-tech and low-pollution industries in their own countries, while shifting high-pollution and high-energy industries to developing countries. The aim of developed countries is to effectively reduce the cost of environmental governance in their countries [9, 10]. Due to stricter environmental regulations, high-pollution, high-energy enterprises in developed countries often voluntarily move to developing countries. Meanwhile, developing countries usually loosen environmental supervision to attract more inflows of FDI—even high-pollution and resource-consuming enterprises—to accelerate the development of their own economy and promote employment [11, 12]. Many studies focus on these issues: For example, Walter and Ugelow suggested that compared with developed countries, developing countries relax their environmental supervision and rely on abundant resources to attract FDI inflows, although some of these industries are highly polluting and resource-consuming [13]. Baumol and Oates found that due to the stricter environmental supervision regulations and measures adopted by developed countries, high-pollution and resource-consuming enterprises are more inclined to move to developing countries to reduce their production costs [14].

The pollution halo hypothesis posits that FDI can bring more advanced production technology and management experience to developing countries. Moreover, through the technology spillover of FDI or through active learning from advanced technology and experience, these host country’s enterprises can improve their energy efficiency, promote low-carbon development, and reduce environmental pollution [15–18]. Some scholars have also conducted theoretical and empirical research on these topics. Mielnika and Goldemberg (2002) conducted an empirical analysis of data from 20 developing countries. The results showed that an increase in FDI can significantly improve the energy utilization of the host country. The direct reason for the increase in energy utilization is that the technology spillover effect of FDI improves the technical level and production efficiency of their enterprises [19]. Li et al. (2016) empirically showed that FDI effectively reduced urban smog pollution in the Pearl River Delta Region [20]. Through empirical research, Xie et al. (2020) found that the spillover effect of FDI on economic development can effectively reduce China’s carbon dioxide concentration and benefit the country’s economic low-carbon growth [21]. Wang and Jin (2002) showed that FDI generally has a relatively higher level of production technology and can reduce environmental pollution and improve energy utilization in the host country by introducing cleaner production technologies and products [22].

In addition to the above views, some scholars argue that FDI does not have a significant impact on the host country’s environmental pollution or energy utilization. Hubler and Keller conducted an empirical analysis of some developing countries, and observed that FDI does not have a direct impact on the energy of developing countries while effectively controlling the various influencing factors of energy utilization [23].

In terms of the impact of FDI on environmental pollution and low-carbon growth of the host country, several scholars believe that the introduction of FDI brings obvious knowledge or technology spillover effects. thereby improving the technological level and production efficiency of the host country. For example, Blackman and Wu analyzed the relationship between FDI quantity and energy utilization in China’s energy industry. They found that an increase in FDI quantity can significantly improve energy utilization and reduce carbon emissions in the host country mainly due to the adoption of the new technology brought in by FDI [24]. Through empirical research on 20 developing countries, Mielnik and Goldemberg found that FDI can replace the traditional production technology of local enterprises via technology spillovers, thereby improving the host country’s technical level and production efficiency, and ultimately promoting their low-carbon development [19]. Qi et al. studied the impact of FDI knowledge spillovers on the energy intensity of different regions in China. The authors found that the impact of FDI on the energy intensity and low-carbon growth of different regions in China are obviously different [25]. Overall, existing scholars have analyzed the different impacts and reasons of FDI on environmental pollution or low-carbon growth in the host country. However, these analyses mainly focus on the macro-level research of the host country, and rarely involve the industry level or the regional level, let alone the impact of FDI on low-carbon growth in high-tech industries or STPs.

### 2.2 FDI and the host country’s level of knowledge and technology accumulation, or the knowledge and technology spillover effect of FDI

The knowledge and technology spillover effects of FDI have been widely studied by scholars globally since the 1960s. In general, FDI can not only make up for domestic funding gaps and promote labor employment, but can also improve the host country’s technical level and innovation ability, thereby promoting economic development and low-carbon growth in the host country [26]. Sha and Sun analyzed the impact of FDI on the innovation capability of high-tech industries in China from the perspective of subdivided industries, and found that FDI has improved the innovation ability of Chinese high-tech enterprises to a certain extent. Moreover, the effect of FDI is related to industry openness. That is, the higher the industry openness, the more significant the knowledge spillover effect. Todo analyzed the knowledge spillover effects of FDI on Japanese domestic companies based on Japanese manufacturing data. The result shows that, unlike the capital stock, the R&D stock of FDI can significantly improve the production efficiency of Japanese companies, implying that the knowledge spillover of FDI is generated through R&D activities rather than production activities [27]. Gilbert analyzed the knowledge spillover effects of FDI based on industrial agglomeration theory and believed that industrial agglomeration is conducive to the acquisition of knowledge spillovers and can effectively improve the innovation performance of enterprises [28].

FDI may promote the technological progress of the host country through knowledge or technology spillover. However, the STPs’ absorption of FDI technology or knowledge spillovers may exhibit significant differences due to their technical level, knowledge accumulation, human resource accumulation, and other factors of the host country. Some scholars have also shown that the knowledge or technology spillover effects of FDI on the host country are subject to different factors and even exhibit nonlinear characteristics. For example, Glass and Alfaro observed that the technological level of enterprises in the host country will significantly affect their absorptive capacity for FDI knowledge spillover. When the technical gap between the enterprise and FDI is small, it can effectively encourage the enterprise to carry out technological imitation and learning, absorb FDI knowledge spillover, and thus improve its own technical level. When this technical gap is large, the absorptive capacity of the STPs is poor, and FDI may negatively impact the host country’s technological progress [9, 10]. Hedong empirically found that the technology spillover of FDI is affected by the threshold variable and knowledge absorptive capacity and presents nonlinear characteristics [29]. Suyanto et al. conducted an empirical study on the technology spillover effects of FDI in Indonesia using plant-level panel data. The authors found that FDI can significantly improve the production efficiency of enterprises, and enterprises with R&D investment will obtain more spillover benefits. This indicates that enterprises with R&D investment have stronger knowledge and technology absorption capacity [30].

Furthermore, the host country’s human capital, knowledge accumulation, or technological level will impact their absorption effect on FDI technology spillovers. Borenszte found that the level of human capital accumulation in the host country directly determines its absorptive capacity for FDI knowledge spillovers, which in turn affects the impact of FDI on the economic low-carbon growth of the host country [31]. Marques found that FDI has different impacts on the pollution emission levels of countries with different income levels through comparative studies. Furthermore, the technology absorptive capacity of middle-income countries has a greater impact on FDI in reducing the host country’s pollution emissions [32].

In summary, FDI has an important impact on the technological progress of the host country. Meanwhile, the absorption effect of the host country on FDI is subject to many factors. Moreover, the capital accumulation, knowledge accumulation, and technical level of the host country also largely affect its absorption capacity for FDI to a greater extent. Therefore, as the host country improves in all aspects, the impact of FDI will show complex nonlinear characteristics. That is, the impact of FDI on the host country’s technological progress and low-carbon growth will show non-linear characteristics due to the difference in its human capital accumulation, knowledge accumulation, or technological level.

### 2.3 STPs’ performance and influencing factors: The impact of FDI

The STPs are the gathering areas of national/regional high-tech enterprises and important platforms to conduct R&D activities and transforming scientific and technological achievements. These activities and achievement transformation represent and influence the level of technological innovation and sustainable development of a country or region. Therefore, there is a strong correlation between STPs and high-tech industries. Several studies focus on the impact of FDI on the host country’s STPs or high-tech industries [33]. For example, Hu examined China’s national STPs and regional economic growth, and observed that FDI has played a significant role in improving the production efficiency of STPs [34]. Liu and Zou analyzed the impact of greenfield FDI on the innovation performance of Chinese high-tech industries. The results show that greenfield FDI has a significant positive impact on the innovation performance of high-tech industries. Furthermore, the greenfield FDI’s R&D activities have spillover effects both within and between high-tech industries [35]. More broadly, Jiang and Xia observed that the competitive effect brought about by FDI is not conducive to China’s innovative development, but it will promote R&D activities in high-tech industries through demonstration effects and the flow of scientific and technological personnel [36]. Regarding the knowledge spillover effects of FDI on STPs or high-tech industries, Jiang and Zhang found that FDI has a positive technological spillover effect on some industries, and the contribution of these technology spillovers to the technological progress of China exceeds that of their own R&D investment in high-tech industries [37]. In terms of the impact of FDI on the R&D of high-tech enterprises, Kathuria analyzed the impact of FDI on medium- and high-tech firms in India during the post-reform period. The results show that FDI had a negative impact on the R&D investment of Indian medium- and high-tech firms in the first phase of 1994–1996, but not during 1999–2001 [38].

In summary, we find that there are relatively fewer studies on the impact of FDI on STPs or high-tech industries, and the focus is mainly on R&D investment, innovation performance, and production efficiency of STPs, rather than on low-carbon development issues.

### 2.4 Theoretical analysis

The existing literature shows that FDI can improve: 1) the innovation ability and technical level of local enterprises to a certain extent, and 2) the host country’s production efficiency and reduce environmental pollution and promote its low-carbon development. However, the local enterprises’ knowledge and technology level may affect the absorption of FDI in the host country and may exhibit nonlinear characteristics. Several studies focus on the impact of FDI on China, and mainly involve economic and social development, environmental pollution, carbon emissions, and energy intensity among others. However, there are few studies on the impact of FDI on STPs. Here, we present the summary and analysis of the theoretical base for this study.

Since the reform and opening-up, China has attracted the highest FDI inflows for several years [39]. FDI has greatly promoted China’s economic and social development via its capital investment, technology introduction, and knowledge spillover, among others [40–43]. Since their establishment, national STPs have undergone rapid development, with increasing knowledge accumulation, improving innovation levels, and decreasing energy consumption intensity. There are two reasons for this: 1) the large investment in R&D and personnel, and 2) factors such as the introduction of FDI and knowledge spillover. FDI positively impacts the technical level of national STPs, improves their production efficiency, and promotes their low-carbon growth.

Meanwhile, knowledge accumulation in STPs also affects the impact of FDI on their low-carbon growth in a nonlinear way. This is because the knowledge accumulation of STPs largely reflects their technology and knowledge absorption capacity. When knowledge accumulation of STPs is at different levels, their technology and knowledge absorption capacity will also differ. When the level of knowledge accumulation of STPs is low, the technological gap between FDI quantity and STPs is relatively obvious. Increasing the FDI quantity can improve the knowledge accumulation and technical level of STPs, improve their production efficiency, and promote their low-carbon growth. As knowledge accumulation in STPs improves, the technological gap between the two gradually narrows. However, the increase in FDI quantity may gradually weaken the impact of FDI on the low-carbon growth of STPs. Nevertheless, from the perspective of FDI quality, improving knowledge accumulation in STPs can improve their knowledge absorption capacity. This is conducive to their absorption of high-quality knowledge spillover from FDI. Therefore, as knowledge accumulation in STPs improves, the impact of FDI quality on the low-carbon growth of STPs will gradually increase.

In summary, the impact of FDI quantity and quality on the low-carbon growth of national STPs will show differentiated nonlinear characteristics. These features will be affected by the level of knowledge accumulation of STPs. The core of this paper focuses on the type of observed nonlinear characteristics.

## 3. Research methodology

### 3.1 Threshold regression method

The impact of FDI on low-carbon development may be affected by the STPs’ knowledge accumulation, that is, the impact has a threshold effect based on the STPs’ knowledge accumulation. To effectively test the significance of this threshold effect and avoid the error caused by manual grouping, this study adopts Hansen’s nonlinear panel threshold regression model to construct a piecewise function with knowledge accumulation of STPs as the threshold variable [44].

$$\{\begin{array}{c} {\text{y}}_{\text{i}}=\beta_{1}\text{x}+\varepsilon_{\text{i}},\;\text{q}\leq \gamma \\ {\text{y}}_{\text{i}}=\beta_{2}\text{x}+\varepsilon_{\text{i}},\text{q}>\gamma \end{array}$$ (1)

Where q represents the threshold variable and γ the threshold value to be estimated. By adding the indicator function I(·), Eq (1) can be expressed by Eq (2) as follows:

$${\text{y}}_{\text{i}}=\beta_{1}{\text{x}}_{\text{i}}\cdot \text{I}({\text{q}}_{\text{i}}\leq \gamma )+\beta_{2}{\text{x}}_{\text{i}}\cdot \text{I}({\text{q}}_{\text{i}}>\gamma )+\varepsilon_{\text{i}}$$ (2)

The single threshold regression model is shown in Eq (3) as follows:

$$\text{lc}2_{\text{it}}=\beta_{0}+\beta_{1}{\text{fdi}}_{\text{it}}\cdot \text{I}({\text{q}}_{\text{i}}\leq \gamma )+\beta_{2}{\text{fdi}}_{\text{it}}\cdot \text{I}({\text{q}}_{\text{i}}>\gamma )+\beta_{\text{j}}{\text{Z}}_{\text{it}}+\varepsilon_{\text{it}}+\mu_{\text{i}}$$ (3)

The double threshold regression model is shown in Eq (4) as follows:

$$\text{lc}2_{\text{it}}=\beta_{0}+\beta_{1}{\text{fdi}}_{\text{it}}\cdot \text{I}({\text{q}}_{\text{i}}\leq \gamma_{1})+\beta_{2}{\text{fdi}}_{\text{it}}\cdot \text{I}(\gamma_{1}<{\text{q}}_{\text{i}}\leq \gamma_{2})+\beta_{3}{\text{fdi}}_{\text{it}}\cdot \text{I}({\text{q}}_{\text{i}}\geq \gamma_{2})+\beta_{\text{j}}{\text{Z}}_{\text{it}}+\mu_{\text{i}}+\varepsilon_{\text{it}}$$ (4)

Where β_0_ denotes the intercept term; β_1_, β_2_, β_3_, and β_j_ are the parameters to be estimated; i represents the STPs; t is the year; Z_it_ represents the series of control variables; I (∙) is an indicative function; q is the threshold variable; γ is the threshold value to be estimated; μ_i_ is the individual fixed effect of the STPs; and ε_it_ is the random interference term.

### 3.2 Variable selection

1. Dependent variable. Combining the research goals and data, this study employs the average comprehensive energy consumption of the industrial added value of 10,000 yuan as the dependent variable to measure the low-carbon development level of national STPs (*lc*). This variable is calculated by converting this average comprehensive energy consumption in terms of standard coal.
2. Explanatory variables. This study analyzes the impact of FDI on the low-carbon development of STPs from the perspective of FDI quantity and quality, which are used as the explanatory variables. FDI quantity (fdi1) is calculated by the annual total FDI of the city where the STP is located. It is relatively difficult to effectively measure FDI quality. This study draws on the calculation method of Wang and Luo [45], as shown in Eq (5) below:
  $${\text{fdi}}_{2}=({\text{FDI}}_{\text{it}}/{\text{FDI}}_{\text{t}})/({\text{GDP}}_{\text{it}}/{\text{GDP}}_{\text{t}})$$ (5)
  Where FDI_it_ represents the FDI amount of the city in which the i^th^ STP is located in period t, and FDI_t_ that of all cities in period t. GDP_it_ denotes the GDP of the city where the i^th^ STP is located in period t, and GDP_t_ the GDP of all cities in period t.
3. Threshold variables. To effectively analyze whether FDI quantity and quality have nonlinear effects on the low-carbon development of STPs under different levels of knowledge accumulation, this study takes knowledge accumulation as a threshold variable. This variable is based on the continuous production of new knowledge. Currently, scholars often adopt patents as an indicator to denote knowledge and technology in empirical analysis [46, 47]. Although this method has shortcomings, the number of patents is still an important key indicator of the output of scientific and technological innovation. Therefore, this study also selects the number of patent applications as an indicator for new knowledge. The number of patent applications rather than patent grants is used as an indicator for two reasons: 1) There is a short time lag from reviewing the patent application to patent licensing. Therefore, the number of patent applications is more appropriate; and 2) The number of patent applications is less affected by human factors in government departments and statistics are more objective.
  This study adopts the perpetual inventory method proposed by Goldsmith using the number of patent applications to calculate the knowledge accumulation of the STPs. The specific equation is as follows:
  $${\text{KA}}_{\text{it}}=(1-\text{d}){\text{KA}}_{\text{i},\text{t}-1}+{\text{P}}_{i,t-1}$$ (6)
  Where KA_it_ denotes the knowledge accumulation of the i^th^ STP at the beginning of period t, KA_i,t−1_ that of the i^th^ STP at the beginning of period t-1, and P_*i*,*t*−1_ the new knowledge produced by the i^th^ STP in period t-1. The knowledge accumulation of the base period is KA_i0_ = P_*i*,0_ · (1 + g_*i*_)/(g_*i*_ + *d*), and g_*i*_ is the average annual growth rate of new knowledge in the i^th^ STP from 2011 to 2018. Following previous literature, this study employs a 15% depreciation rate for calculation.
4. Control variables. Combining previous literature and data sources, this study selects five controlled variables from the perspective of the STP and the city where it is located to limit errors. The indicators based on the STP include the proportion of college-graduated personnel and the intensity of R&D investment. The former refers to the proportion of personnel with a college degree or above in the STP (*cd*), and the latter is the ratio of R&D expenditure to the operating income of the year (*rd*). City-based indicators include three items: urban environmental regulation (*er*), urban scientific and technical level (*ust*), and real per capita GDP (*pgdp*). This paper borrows the entropy method from Feng et al. to calculate urban environmental regulation indicators: the utilization rate of industrial solid waste, the removal rate of flue gas, the removal rate of SO_2_, the compliance rate of waste effluent, and the harmless treatment rate of live garbage in the city in which the STP is located [48]. The urban technology level is obtained by dividing the urban annual scientific and technological expenditure by the annual total expenditure. The urban economic level is measured by the city’s GDP per capita.

### 3.3 Data sources and descriptive statistics

The panel data in this study are derived from the China Torch Statistical Yearbook, China City Statistical Yearbook, and some are provided by the Torch High-tech Industry Development Center of the Ministry of Science and Technology of China. The data cover the average comprehensive energy consumption per 10,000 yuan of added value of enterprises, the number of annual patent applications, the number of college personnel, the amount of FDI investment, and the GDP of the cities in which China’s 52 STPs are located.

The descriptive statistics of each variable are shown in Table 1. There is still a large gap between the maximum and minimum values of lnlc, and the standard deviation is also relatively large. This indicates that there are large differences in the low-carbon development level of national STPs in different regions. Furthermore, STPs with relatively low levels of low-carbon development have greater potential for improvement. Meanwhile, there is also a large gap between the maximum and minimum values of lnka, and the standard deviation is also relatively large. This indicates that there are large differences in the level of knowledge accumulation in national STPs in different regions. Furthermore, the large gap in the level of knowledge accumulation will also have a significant threshold effect on the impact of FDI on the low-carbon development of STPs. Moreover, there are also large gaps between the maximum and minimum values of both FDI quantity and quality, and the standard deviations are also relatively large. This indicates that there are large differences in FDI quantity and quality in the cities in which the national STPs are located. Further, the impacts on the low-carbon development of STPs may differ. Overall, the average value and standard deviation of the variables adopted in this study are normal and have a certain degree of dispersion, which can meet the requirements of regression analysis.

**Table 1:** Descriptive statistics of variables.

Variable	Number of Samples	Average Value	Minimum Value	Maximum Value	Standard Deviation
lnlc	416	-1.309	-3.764	1.952	1.039
lnfdi1	416	11.843	6.328	14.941	1.453
lnfdi2	416	-0.484	-5.139	1.227	0.942
lnka	416	7.649	4.486	11.773	1.295
lncd	416	-0.735	-2.758	0.046	0.317
lnrd	416	-3.616	-5.442	-2.649	0.411
lner	416	-0.272	-1.279	-0.037	0.155
lnust	416	-3.822	-5.965	-1.816	0.664
lnpgdp	416	11.081	9.666	13.056	0.501

## 4. Results and discussion

### 4.1 Multiple collinearity test

This study uses the Variance Inflation Factor (VIF) to test for multicollinearity. The results are shown in Table 2. The VIF value of FDI quantity is the highest but still less than 10. The VIF values of other explanatory variables are also less than 10, indicating that there is no multicollinearity between the explanatory variables used in this study.

**Table 2:** VIF multicollinearity test results.

Variable	VIF	1/VIF
fdi1	2.33	0.428
fdi2	1.95	0.514
ust	1.57	0.638
pgdp	1.56	0.641
rd	1.55	0.645
cd	1.41	0.707
er	1.16	0.859
Mean	1.6	0.633

### 4.2 Threshold effect significance test

**Fig 2:**
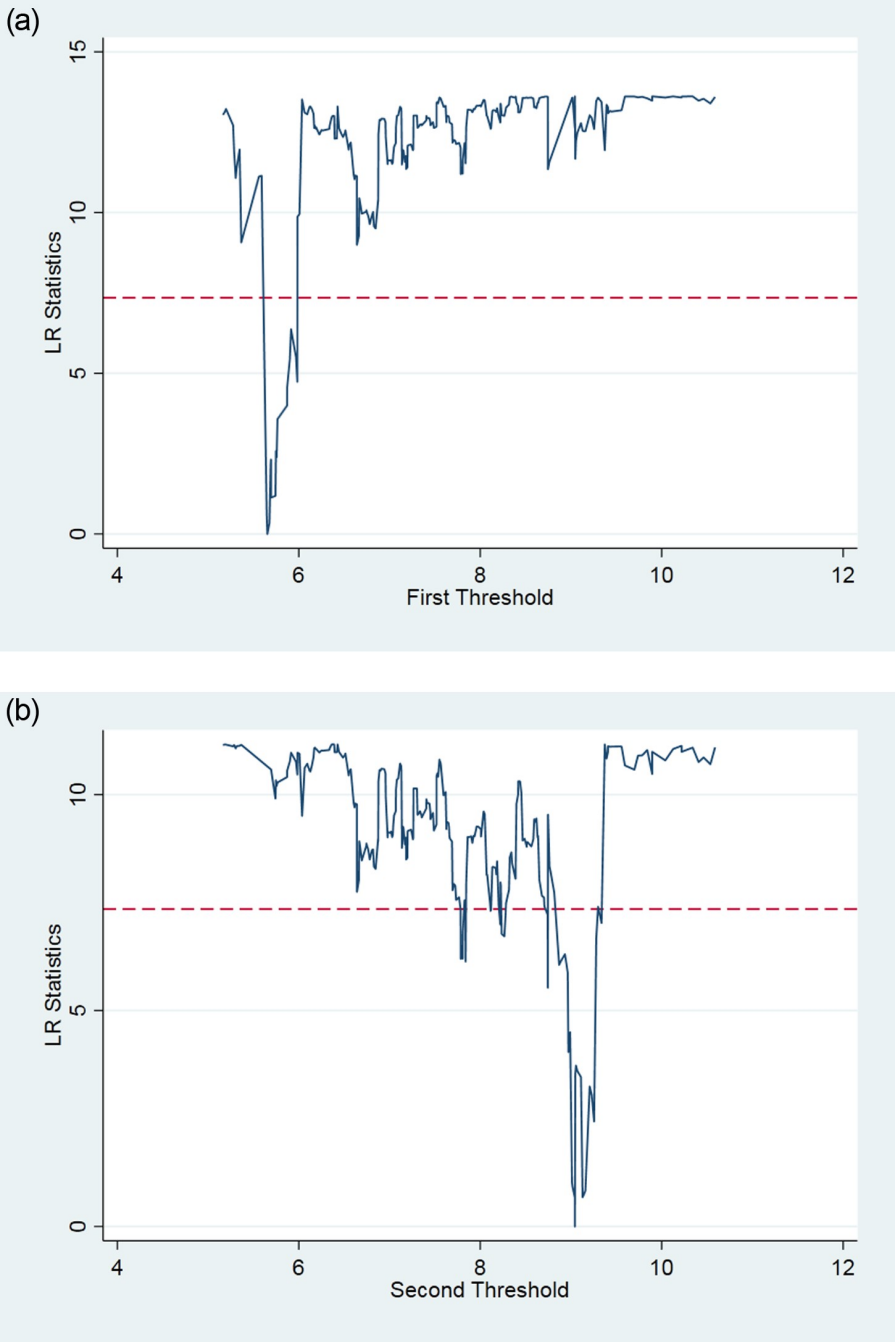
a. The LR map corresponding to the first threshold estimate of FDI quantity. b. The LR map corresponding to the second threshold estimate of FDI quantity.

**Fig 3:**
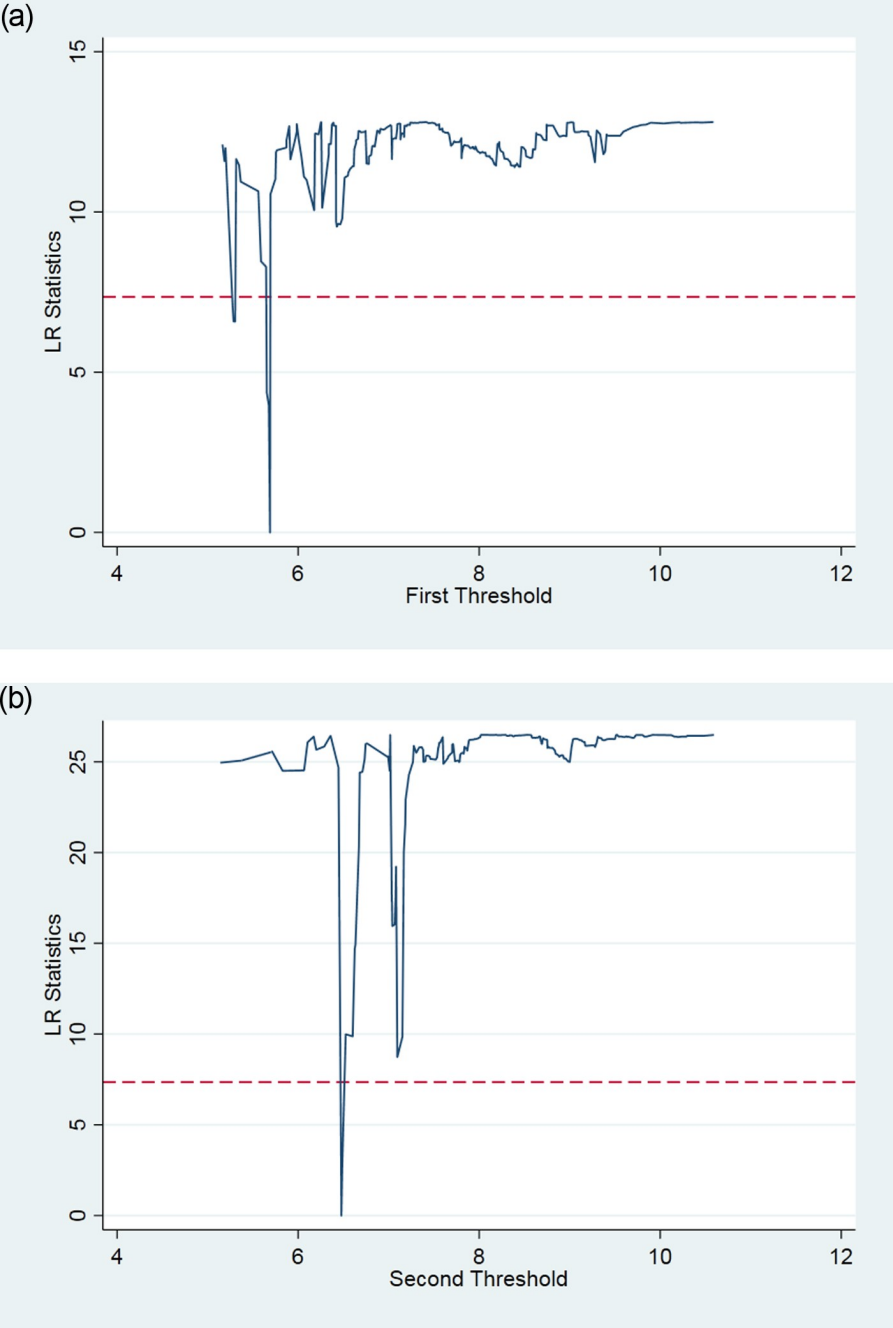
a. The LR map corresponding to the first threshold estimate of FDI quality. b. The LR map corresponding to the second threshold estimate of FDI quality.

To test whether the impact of FDI quantity and quality on low-carbon growth has a threshold effect based on the knowledge accumulation of STPs, and whether there are multiple threshold effects, this study verifies the threshold effect of knowledge accumulation for FDI quantity and quality. The significance test results of the threshold effect of FDI quantity and quality using a threshold effect test with knowledge accumulation as the threshold variable are shown in Tables 3 and 4. The tables show the F value and the corresponding self-sampling P value and the critical values at the 1%, 5%, and 10% significance levels. The results show that the single and double thresholds of FDI quantity are significant at the 10% and 1% levels, respectively. Meanwhile, the single threshold of FDI quality fails the significance test, and the double threshold is significant at the 1% level. The likelihood ratio function graphs of the FDI quantity and FDI quality are shown in Figs 2a, 2b, 3a and 3b, respectively. Together, this evidence, according to Hansen’s (1999) threshold theory, suggests that the impact of FDI quantity and quality on the low-carbon growth of STPs has a threshold effect based on knowledge accumulation.

**Table 3:** FDI quantity threshold significance test and confidence intervals.

Threshold Variable	Model	F-value	P-value	0.010	0.050	0.100	Threshold Estimates	0.95 Confidence Interval
FDI1	Single threshold	29.71*	0.087	56.814	48.18	41.7835	7.022	[6.872,7.082]
	Double threshold	51.263***	0.000	36.6567	29.8153	25.6673	5.654	[5.618,5.977]
							9.044	[8.930,9.048]

**Table 4:** FDI quality threshold significance test and confidence intervals.

Threshold Variable	Model	F-value	P-value	0.010	0.050	0.100	Threshold Estimates	0.95 Confidence Interval
FDI2	Single threshold	11.481	0.297	53.286	41.4553	35.9355	6.127	[6.120,6.146]
	Double threshold	43.975***	0.003	35.8713	25.1137	22.2444	5.689	[5.661,5.693]
							6.478	[6.446,6.486]

### 4.3 Threshold effect test

The study adopts Hansen’s nonlinear panel threshold regression model for quantitative analysis, and the results are shown in Tables 5 and 6. First, as shown in Table 5, when the level of knowledge accumulation is below threshold 1, the FDI quantity has a significant negative impact on energy consumption. That is, an increase in the FDI quantity can effectively reduce the unit energy consumption, thereby promoting the low-carbon development of the STPs. When the level of knowledge accumulation exceeds threshold 1 but does not reach threshold 2, although the FDI quantity has a significant negative impact on the energy consumption, its impact coefficient (the marginal effect of the impact) shows a gradual decline. That is, the impact of FDI quantity on the low-carbon development of the national STPs weakens. The reason may be that when knowledge accumulation is below threshold 1, the independent innovation capability, the overall technological capability, and production efficiency of the STPs are relatively low. Therefore, due to the existence of knowledge and technology gaps between the STP and FDI in this period, the increase in the FDI quantity can bring in more advanced technology, management experience, and technology spillover effects. These effectively improve the independent knowledge innovation capabilities and technical level of the STPs’ enterprises, and ultimately, improve their technical and production efficiency, and promote the low-carbon development of STPs. However, when the level of knowledge accumulation of STPs is higher than threshold 1 and lower than threshold 2, and with the gradual improvement of knowledge accumulation, the technological capabilities and production efficiency of STPs greatly improve. Then, the dependence of national STPs on advanced technology, management experience, and knowledge spillover effects brought about by the increase in FDI quantity gradually decreases. That is, the gap in knowledge and technology between the STP and FDI is reduced so that the influence of FDI quantity on the low-carbon development of STPs gradually weakens.

**Table 5:** FDI quantity threshold model estimation results.

Explanatory Variable	Single Threshold	Explanatory Variable	Double Threshold
lnrd	-0.222 (0.253)	lnrd	-0.245 (0.252)
lncd	-0.094** (0.040)	lncd	-0.115*** (0.041)
lner	-0.097 (0.337)	lner	-0.165 (0.336)
lnust	-0.153 (1.256)	lnust	-0.296 (1.251)
lnpgdp	-0.005 (0.119)	lnpgdp	-0.042 (0.120)
fdi1(ka ≤ 7.022)	-0.584*** (0.173)	fdi1(ka ≤ 5.654)	-0.673*** (0.176)
fdi1(ka > 7.022)	-0.022 (0.017)	fdi1(5.654 < ka ≤ 9.044)	-0.060** (0.024)
		fdi1(ka > 9.044)	-0.019 (0.017)
_cons	1.278*** (0.341)	_cons	1.525*** (0.356)

**Table 6:** FDI quality threshold model estimation results.

Explanatory Variable	Single Threshold	Explanatory Variable	Double Threshold
lnrd	-0.280 (0.254)	lnrd	-0.211 (0.255)
lncd	-0.103** (0.040)	lncd	-0.101** (0.040)
lner	-0.111 (0.338)	lner	-0.083 (0.334)
lnust	-0.251 (1.258)	Lnust	-0.195 (1.253)
lnpgdp	-0.016 (0.117)	lnpgdp	-0.020 (0.117)
fdi2(ka ≤ 6.127)	-0.466*** (0.155)	fdi2(ka ≤ 5.689)	-0.188 (0.204)
fdi2(ka > 6.127)	-0.185** (0.082)	fdi2(5.689 < ka ≤ 6.478)	-0.717*** (0.199)
		fdi2(ka > 6.478)	-0.217*** (0.083)
_cons	1.464*** (0.351)	_cons	1.419*** (0.347)

When the level of knowledge accumulation exceeds threshold 2, the impact of FDI quantity on energy consumption is no longer significant, indicating that it no longer significantly promotes the low-carbon development of STPs. The reason may be that with the massive increase in the level of knowledge accumulation, the independent innovation ability and level of enterprises in the STPs have been continuously enhanced. This indicates that enterprises can make efforts and play an important role in reducing energy consumption and promoting low-carbon development with their own innovation capabilities and production efficiency levels. This also means that as the technological gap between STPs and FDI is getting smaller, the technology spillovers for FDI in the host country will gradually decrease [49–51]. The dependence of the STP on FDI in terms of technology then gradually decreases. As a result, the impact of FDI quantity on the low-carbon development of the national STPs is no longer significant.

Second, from the perspective of FDI quality, when the level of knowledge accumulation is below threshold 1, FDI quality has no significant impact on the STP’s low-carbon development. When the level of knowledge accumulation exceeds threshold 1, FDI quality has a significant negative impact on energy consumption. That is, it can significantly promote the low-carbon development of these STPs. This is because when knowledge accumulation is low, the lack of knowledge and skills leads to low absorptive capacity and innovation ability of the enterprises in STPs. These enterprises are unable to effectively acquire and master FDI knowledge and technology. This situation further results in their inability to enhance their own technological and innovation capabilities. Thus, it becomes impossible to reduce the unit energy consumption of STPs and promote their low-carbon development.

When the level of knowledge accumulation exceeds threshold 1, these enterprises already have abundant knowledge and technical reserves, and independent innovation capabilities, can effectively acquire high-quality FDI knowledge and technology, and can transform them into their own knowledge and innovation capabilities. That is, with the improvement of knowledge accumulation of STPs, the FDI quality can improve the technical and production efficiencies of local enterprises through knowledge spillover, thereby significantly reducing their energy consumption and promoting the low-carbon development of STPs.

Meanwhile, when the level of knowledge accumulation exceeds threshold 2, although the FDI quality can significantly reduce the energy consumption and promote the low-carbon development of the STPs, its impact coefficient (marginal effect) shows a gradual decline as knowledge accumulation continues to increase. The reason is that with the further improvement of knowledge accumulation and the independent innovation ability of these enterprises, their technical and production efficiencies have also improved. As a result, the marginal effect of FDI knowledge spillover declines relatively, and the effect of FDI quality on the low-carbon development of these enterprises in STPs gradually declines. This also clearly shows that with the continuous enhancement of independent innovation capabilities of the STPs, their technical and production efficiencies, and ability to promote low-carbon development are increasing day by day, and the dependence on FDI is gradually decreasing.

### 4.4 Discussion

The above empirical results show that both the FDI quantity and quality have an important impact on the low-carbon development of STPs in China. Overall, the continuous increase in knowledge accumulation of high-tech enterprises has gradually improved their independent innovation capabilities. The dependence on FDI has gradually declined, thus weakening the impact of FDI quantity and quality on the low-carbon development of China’s STPs. Therefore, from the perspective of FDI, both the FDI quantity and quality have shown gradual weakening trends in promoting the low-carbon development of STPs. Especially for the FDI quantity, when the level of knowledge accumulation exceeds threshold 2, its impact is no longer significant. The reasons are as follows. First, in the initial period of opening up, the level of knowledge accumulation of STPs was low. The introduction of FDI, especially high-quality FDI, improved the innovation level, technical capabilities, and production efficiency of STPs, thereby promoting low-carbon development in China. Second, as China continues to increase investment in R&D resources and promotes knowledge accumulation, the independent innovation capabilities of STPs are also improving. The technological and knowledge dependence of STPs on FDI has gradually declined, indicating that the role of FDI in promoting the low-carbon development of China has gradually declined. Meanwhile, the independent innovation capabilities of STPs have been increasing.

This research result is in line with the policy orientation of the Chinese government. In the early stages of reformation and opening up, Chinese enterprises were relatively poor in knowledge accumulation, technology and production efficiencies, and independent innovation capabilities. To promote the development of both the economy and the technology, the Chinese government has actively attracted more FDI inflows through a variety of preferential measures, including tax incentives and land approval. Due to the large number of preferential policies, good open environment, and cheap labor, FDI has maintained a rapid growth trend in China for many years. This has played an important role in promoting the economic and social development of China, such as increasing labor employment and promoting technological progress. However, with the continuous improvement of China’s economic development level and independent innovation capabilities, the impact of FDI has gradually declined. In particular, the spillover effect of knowledge and technology has shown a clear downward trend, along with several negative influences, such as environmental pollution and homogeneous competition problems. The Chinese government has also gradually changed its policies on FDI: on the one hand, it actively attracts high-tech FDI to build R&D centers in China, while on the other hand, it no longer provides FDI with super-national treatment. These changes reflect that with the development of the economy and society, China is more inclined to attract high-quality FDI, while effectively controlling the FDI quantity to reduce their negative influences.

## 5. Conclusions and policy recommendations

This study uses knowledge accumulation as a threshold variable and adopts Hansen’s nonlinear panel threshold regression model to analyze the impact of FDI quantity and quality on the low-carbon development of STPs in China. The results show the following. First, both the FDI quality and quantity have a significant impact on the low-carbon development of STPs. Their effects show nonlinear characteristics with changes in the level of knowledge accumulation in the STPs. Second, when the level of knowledge accumulation is below threshold 1, the FDI quantity significantly and positively affects the low-carbon development of STPs. That is, FDI quantity can promote the low-carbon development of STPs When the level of knowledge accumulation is higher than threshold 1 and lower than threshold 2, the positive impact of FDI quantity gradually decreases but is still significant. When the level of knowledge accumulation exceeds threshold 2, the impact of FDI quantity is no longer significant. Third, when the level of knowledge accumulation is below threshold 1, the FDI quality has no significant impact on the low-carbon development of STPs. When the level of knowledge accumulation is higher than threshold 1 and lower than threshold 2, the FDI quality has a significant positive impact. That is, it can significantly promote the low-carbon growth of STPs. When the level of knowledge accumulation exceeds threshold 2, the positive impact of FDI quality gradually decreases.

Based on the above conclusions, this paper proposes the following recommendations. First, different FDI introduction strategies should be adopted for the different development levels of STPs with different levels of knowledge accumulation. Due to the distinct development levels of national STPs in different regions, the impact of FDI quantity and quality on the low-carbon development of national science parks differs. Therefore, different orientations of FDI introduction strategies should be implemented according to the development level and knowledge accumulation level of national STPs in different regions. For example, in economically developed areas or cities with high levels of knowledge accumulation, a high-quality policy that actively introduces high-quality FDI and controls FDI quantity is advisable. This will help reduce the negative effects of FDI quantity. However, in underdeveloped areas or cities with relatively weak knowledge accumulation levels, it is necessary to attract more FDI inflows regardless of FDI quality to achieve sustainable economic and social development.

Second, R&D investment in high-tech enterprises and improvements in the knowledge accumulation level of legal personnel in STPs should be strengthened. The level of knowledge accumulation in national STPs largely affects and determines their level of independent innovation. This has a direct and important impact on their low-carbon growth. Hence, the Chinese government is advised to further strengthen its policy and financial support for the STPs. This includes improving the basic conditions for innovative development such as public technology platforms, key laboratories, and inspection and testing platforms in STPs. The effective combination efficiency of various innovative elements needs to be further improved, along with knowledge accumulation in the STPs. This will drive the technical level and production efficiency, and promote the low-carbon development of STPs.

Third, the absorption capacity of STPs for high-quality talents should be enhanced, along with improving their independent innovation ability and level. Talents, especially high-quality talents, are key to improving the independent innovation level of STPs. The Chinese government should adopt preferential policies and more funds to promote the introduction of high-level talent in STPs, and strengthen the cultivation and training of technical talents. By improving the quality of talents and optimizing their structure, the level of R&D and innovation of STPs is improved, thereby improving their production efficiency. The improvement of the production efficiency of STPs has promoted their innovation level and technological progress, and ultimately accelerated the realization of the economic low-carbon growth in China.
